# Feasibility analysis on the construction of a web solution for hydrometeorological forecasting considering water body management and indicators for the SARS-COV-2 pandemic

**DOI:** 10.1186/s42467-021-00011-0

**Published:** 2021-10-01

**Authors:** José Roberto Dantas da Silva Júnior, Rizzieri Pedruzzi, Filipe Milani de Souza, Patrick Silva Ferraz, Daniel Guimarães Silva, Carolina Sacramento Vieira, Marcelo Romero de Moraes, Erick Giovani Sperandio Nascimento, Davidson Martins Moreira

**Affiliations:** 1 Manufacturing and Technology Integrated Campus, SENAI CIMATEC, Salvador, BA Brazil; 2 Renewables Energies Campus, Pampa’s Federal University, Bagé, RS Brazil

**Keywords:** Water resources, Hydrometeorological forecast, WRF-Hydro, Artificial Intelligence, SARS-CoV-2 (COVID-19), MATOPIBA

## Abstract

The current scenario of a global pandemic caused by the virus SARS-CoV-2 (COVID19), highlights the importance of water studies in sewage systems. In Brazil, about 35 million Brazilians still do not have treated water and more than 100 million do not have basic sanitation. These people, already exposed to a range of diseases, are among the most vulnerable to COVID-19. According to studies, places that have poor sanitation allow the proliferation of the coronavirus, been observed a greater number of infected people being found in these regions. This social problem is strongly related to the lack of effective management of water resources, since they are the sources for the population's water supply and the recipients of effluents stemming from sanitation services (household effluents, urban drainage and solid waste). In this context, studies are needed to develop technologies and methodologies to improve the management of water resources. The application of tools such as artificial intelligence and hydrometeorological models are emerging as a promising alternative to meet the world's needs in water resources planning, assessment of environmental impacts on a region's hydrology, risk prediction and mitigation. The main model of this type, WRF-Hydro Weather Research and Forecasting Model), represents the state of the art regarding water resources, as well as being the object of study of small and medium-sized river basins that tend to have less water availability. hydrometeorological data and analysis. Thus, this article aims to analyze the feasibility of a web tool for greater software usability and computational cost use, making it possible to use the WRF-Hydro model integrated with Artificial Intelligence tools for short and medium term, optimizing the time of simulations with reduced computational cost, so that it is able to monitor and generate a predictive analysis of water bodies in the MATOPIBA region (Maranhão-Tocantins-Piauí-Bahia), constituting an instrument for water resources management. The results obtained show that the WRF-Hydro model proves to be an efficient computational tool in hydrometeorological simulation, with great potential for operational, research and technological development purposes, being considered viable to implement the web tool for analysis and management of water resources and consequently, assist in monitoring and mitigating the number of cases related to the current COVID-19 pandemic. This research are in development and represents a preliminary results with future perspectives.

## Introduction

The management of water resources is still a global challenge. The latest United Nations Water Development Report [1] highlights that only 10% of developing countries have water quality monitoring systems. Thus, advances in the management of water bodies enhance the performances of these systems, which are valuable tools for the economy and society. In this sense, the current scenario of a global pandemic caused by SARS-CoV-2 highlights the importance of water studies for sewage systems, which in turn are related to major challenges, such as basic sanitation. The survival of Coronaviruses in Water and Wastewater have been reported before the Sar-Cov-2 outbreak. Gundy [2] reported in 2008 coronaviruses surviving upon 10 days in water at 23°C and up to 100 days in water at 4 °C. Recently, regarding to the SARS-CoV-2, studies have found that the virus survival is strongly dependent of water properties, such as temperature, pH, the presence of organic matter and the presence of disinfectant, in case of treated water. Nonetheless, according to Bilal [3] work, is was found SARS-CoV-2 could survive from few days to weeks. Mandal [4] also noticed thar the virus can survive from 1.57 day up to up to 14 days. Additionally, Tran [5] also reported that, the virus could survive over 7 days in water at 23 °C. These findings highlighted virus survival in water and wastewater can vary significantly, especially on different water conditions.

The study by [6] indicated that municipalities with poor sanitation are similarly situated, with the highest numbers of infections and deaths caused by SARS-CoV-2 infection. Particularly, this social problem is related to the management of water resources. Thus, to face these challenges, computational modeling emerges as a valuable mechanism for decision making. However, hydrometeorological representation requires a model capable of characterizing the hydrology of the Earth's surface-atmosphere interface, where measurements and data availability are fundamental for understanding watershed dynamics [7].

In this context, the hydrological modeling module of the WRF model*,* called the WRF-Hydro system, is a state-of-the-art model used in water resources and provides coupling between an atmospheric model and a hydrological model. The WRF-Hydro system was developed by NCAR in partnership with NASA to model and simulate rainfall, reservoir management, and flood forecasting, and it allows users to create, save, and compare future scenarios. The numerical and computational structure of the model allows more details to be obtained [7]. Consequently, the WRF-Hydro model has been applied worldwide in studies of flood forecasting and simulation, severe precipitation events caused by hurricanes, flow simulations in water bodies, soil moisture, evapotranspiration precipitation, and in further studies of hydrometeorological conditions in both arid and humid regions [8–15]. For Brazil, we note the work of [16], who used the WRF-Hydro system in the state of Pernambuco to develop a management tool to simulate rainfall, reservoir management, and flood forecasting. In [17], they also employed the WRF-Hydro in the northeastern region of Brazil, called MATOPIBA, to simulate the flows of water bodies in the region and developed a web tool for application of the model.

The WRF-Hydro model has become a major ally in water resource management and hydrometeorological modeling, as demonstrated in [18]. This model has great potential for studying small and medium hydrographic basins that tend to have lower data availability and fewer hydrometeorological stations. Recently, the authors in [19, 20] implement the WRF-Hydro in order to investigate the potential of the hydrometeorological model coupled in the operational prediction of floods and floods in hydrographic basins in Greece and USA, respectively, demonstrating the good capacity of the model in reproduce the flow observed during flood episodes. In East Africa, [14] applies the WRF-Hydro modeling system to better understand the hydrometeorological conditions of the Tana River basin in Kenya. The study investigates precipitation, evapotranspiration and soil water infiltration, quantifying the atmospheric-terrestrial water balance in this region. In [21] the authors simulate the spatial distribution of soil moisture in hyper arid environments such as the UAE. The work in [22] estimates the flow of the Brahmaputra River located between India and Bangladesh, using the WRF-Hydro model. The results show good correlation between simulated and observed data. Notably, the workflow for this system is based on preprocessing, processing, and postprocessing phases. These steps require handling large amounts of input data with a high spatial resolution, such as topography, land use, and occupation, as well as information from lakes and rivers, among others, which results in many datasets needing to be analyzed. Additionally, the model requires a robust computer structure for processing.

These WRF-Hydro requirements often limit its application, as the computer structure is not an easily accessible resource, especially in Brazil. Surface data at high resolution and data measured for model validation are not always available. Because of these computational difficulties, there is the alternative possibility of applying artificial intelligence (AI) techniques to facilitate processing of WRF-Hydro, which could improve the model predictions and postprocessing, as it is possible to train the network with the measured data; additionally, considering the history of the WRF-Hydro model, forecasts can be obtained more quickly with fewer computational costs. At this point, it is important to point out that AI has already been applied in the WRF model without coupling of the water module, as in [23–28], to improve the modeled results and predictability of future data. However, the application of AI together with WRF-Hydro is still under development, as in the studies of [29, 30]. A major advantage expected in the use of AI to facilitate the processing and postprocessing of WRF-Hydro is the lower demand for computational resources and greater speed in forecasting water body flows when the network is trained.

Faster forecasting with the use of AI will help manage and better characterize water resources. In the SARS-CoV-2 pandemic period, indicators, such as levels of contamination in wastewater/water bodies by SARS-CoV-2, can identify regions that are more contaminated by the virus, as exposed by [31]. In Brazil, unfortunately, much of the domestic sewage is added directly to water bodies. Approximately 54% of Brazil's sewage is collected, and only 69% of the sewage collected is treated [32]; consequently, the SARS-CoV-2 virus is also released into water bodies. Studies such as [33–35] identify the virus in wastewater, and the work developed in Belo Horizonte, Brazil [36] also identified the virus in the sewage system. The paper of [31] also highlights that the monitoring of wastewater is a good indicator of SARS-CoV-2 contamination levels. However, as shown in [34], laboratory tests are not sufficient to predict the increase or decrease in the level of infection.

Additionally, the [37] hydrological variables (lake area, river length, precipitation, and volume of water resources) were related to the number of cases confirmed to be SARS-CoV-2. The authors [37] concluded that hydrological variables have a significant correlation with the prediction of SARS-CoV-2 incidences, suggesting that water transmission of SARS-CoV-2 is a source of virus spread and may pose a threat to public health. However, [38] reported that the transmission of SARS-CoV-2 through contact with contaminated waters and contaminated feces is uncertain and needs further study.

The study [21] emphasized the use of epidemiological tools, such as wastewater-based epidemiology (WBE), to assist in water quality management, using SARS-CoV-2 markers in wastewater to trace the profile of contamination in cities and assist in mitigating disease outbreaks. Notably, even though it is an important practice, monitoring water bodies requires sampling campaigns and specific laboratory analyses to determine the level of water contamination, which can be costly and requires the training of personnel. Given these difficulties, the application of WRF-Hydro coupled with AI can serve as an auxiliary rapid response tool to understand the dynamics, displacement, and reach of SARS-CoV-2 in water bodies, thereby assisting in the management of both water resources and the pandemic.

Thus, this study aims to analyze the feasibility of a computational tool for the simulation, monitoring, and analysis of water predictability using WRF-Hydro and AI in the MATOPIBA region, which is the acronym of the states of Maranhão (MA), Tocantins (TO), Piauí (PI) and Bahia (BA), where the management of water resources is of strong socioeconomic interest for the Brazilian government.

## Methodology

### Study area

The states that comprise the MATOPIBA region have a large volume of the freshwater available in Brazil, especially the Northwest Atlantic, Parnaíba, Tocantins-Araguaia, and São Francisco basins.

The state of Maranhão is in northeastern Brazil, and its vegetation covers the Amazon forest, savanna, and caatinga. The Tocantins is located in the central portion of Brazil, but officially is part of northern region, bounded to the west by the Araguaia River and in the center by the Tocantins River, with an economy comprising mostly agriculture [39–42]. Piauí is located in northeastern Brazil, and its economy is focused on industry (chemistry, textiles, beverages, agriculture, and livestock). Bahia is also in the northeast region, and its economy is composed of agriculture, industry, mining, tourism, energy (oil and gas and renewables), and services [39–42].

The scenario presented involves a region with great agricultural quality that requires the exploitation of land use and water resources, and in turn, the adjacent regions are inhabited by people who comprise the region's workforce; additionally, these people utilize the available water resources in the region. Therefore, evaluating the feasibility of a tool for monitoring and analyzing water predictability to manage these resources and forecasting droughts/floods, in addition to verifying the level of contamination by SARS-CoV-2 in the region, is extremely important.

### Hydrometeorological modeling with WRF-Hydro system

Simulations were performed using the WRF-Hydro hydrometeorological modeling system. The coupled model included a high-resolution hydrological model and a thin-scale weather model within a single system. This reduces the uncertainties associated with the spatial distribution and precipitation volume, demonstrating adequate predictive potential for surface runoff, flow forecasting, and flooding.

**Fig. 1:**
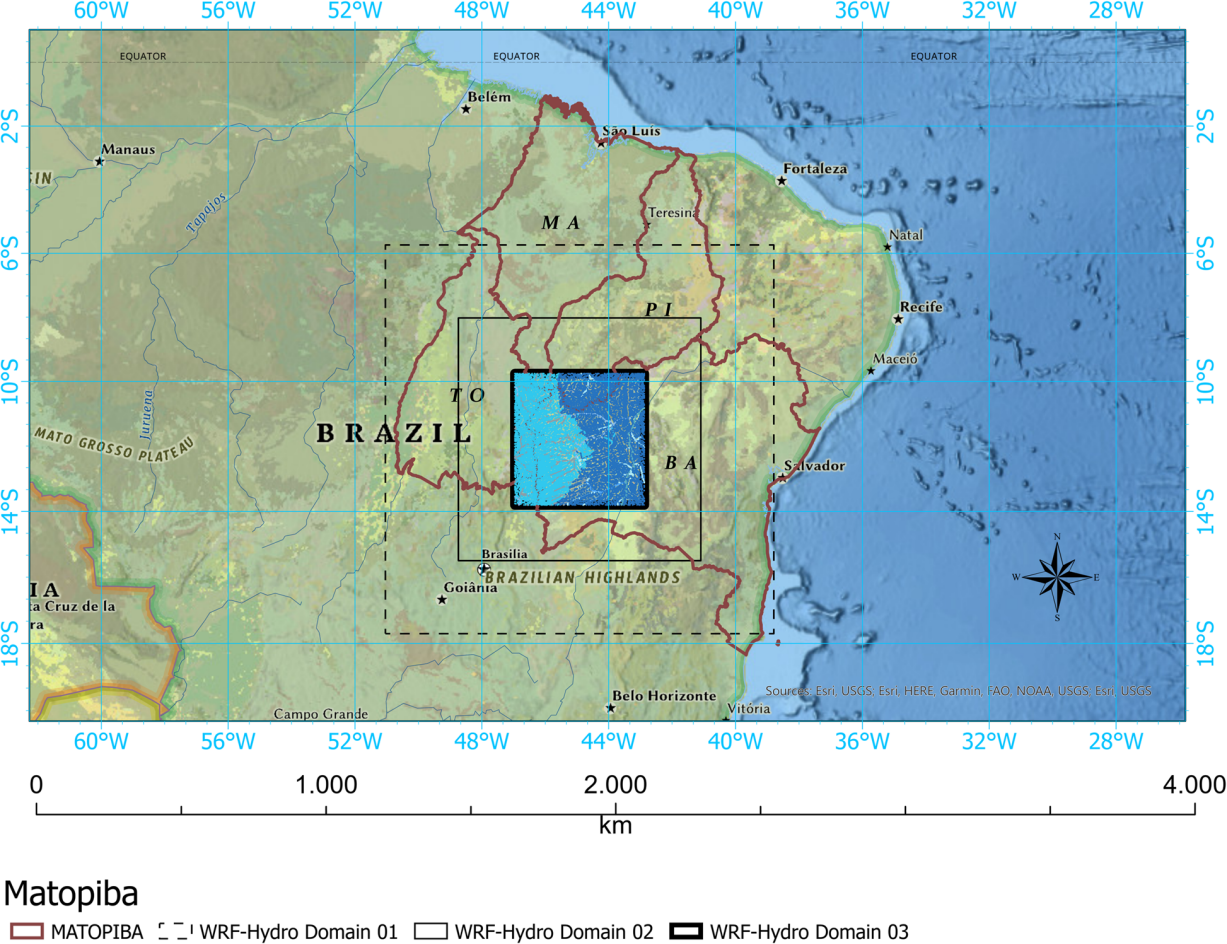
Study area

**Fig. 2:**
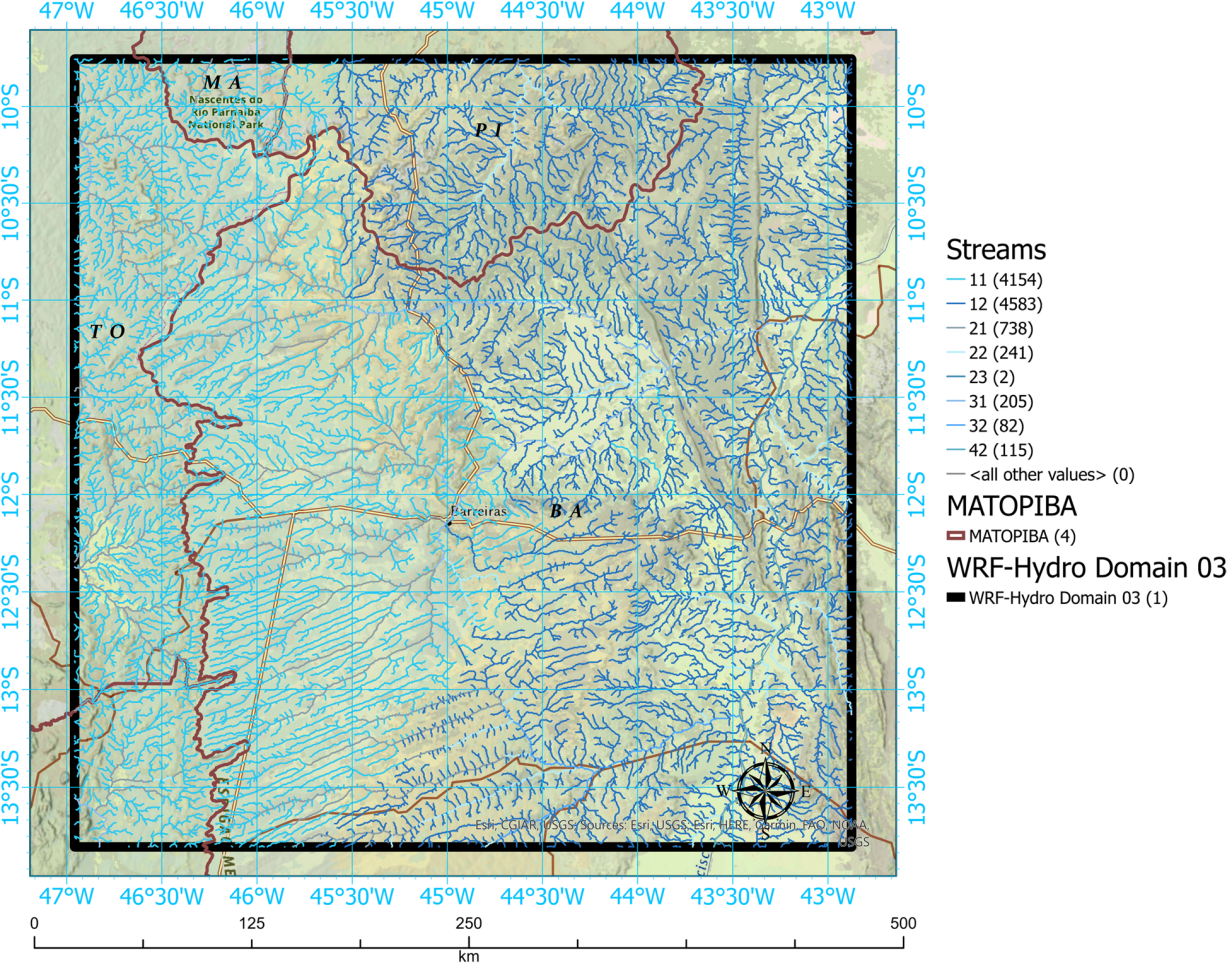
Location of the domain of interest with water flows

The modeling was divided into two parts (meteorological and hydrological). The weather model was configured in three nested degrees with resolutions of 9 km, 3 km, and 1 km, according to Fig. 1. Figure 2 D03 of the WRF-Hydro system.

The domain of interest (D03) has a horizontal resolution of 1 km in a category of 448 x 454 cells, which included geoprocessing tools for resizing the grade to 250 m in terms of water scope. An overview of the spatial settings is shown in Table 1

**Table 1:** Configuration of domains

Domain	D01	D02	D03
Horizontal Resolution	9 km	1.6 km	1 km
Number of Cells	150x150	280x280	448x454
Domain Size	1350x1350 km	840x840 km	448x454 km

The simulation was performed using the WRF-ARW core version 3.9.1 with initialization at 12 PM (UTC) on March 10, 2019, extending until 18 PM (UTC) on March 20, 2019 (246 h of simulation). This simulation aimed to generate the preliminary data necessary for geoprocessing in the ArcGIS application. The initial and contour conditions used in the simulations came from the global atmospheric model GDAS-FNL *(Global Data Assimilation System Final Analysis)* of NCEP (National Centers for Environmental Prediction*),* with a horizontal resolution of 0.25° x 0.25° and a temporal resolution of 6 hours. Land use and occupation topography data were provided by the United States Geological Survey (USGS), and these data have a temporal resolution of 30 seconds [43].

**Fig. 3:**
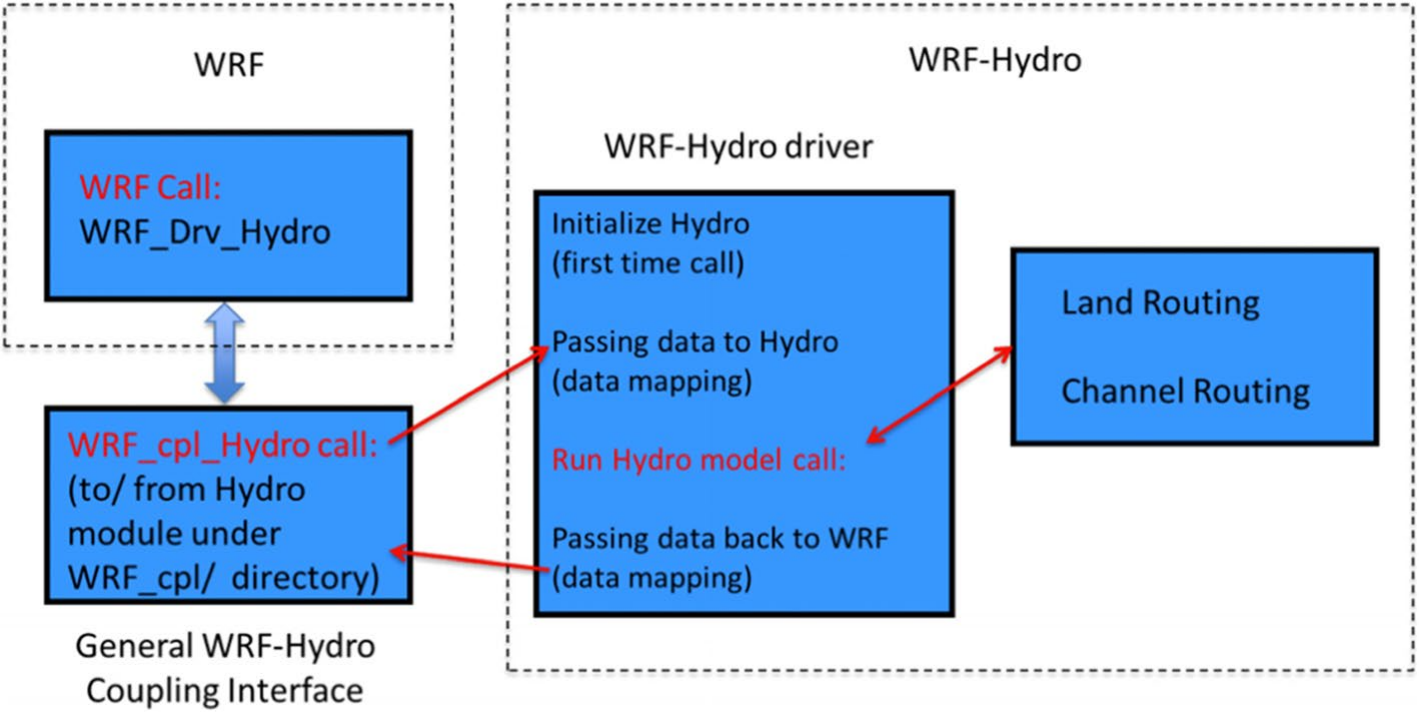
Structure of the WRF-Hydro system given the physical aspects related to meteorology and the type of coupling. Source: An adapted figure of [7]

A growing trend in the use of unidirectional or two-way hydrometeorological modeling systems indicates the importance of integrated atmospheric, geological, and hydrological modeling tools. Typical studies use the WRF model and hydrological models, such as the WRF-Hydro model [18]. Due to the robust architecture, the WRF-Hydro system is state-of-the-art in hydrometeorological modeling, whether in uncoupled or coupled mode. Figure 3 shows the representation of how models are integrated.

After preprocessing with WRF, as shown in Fig. 3 the intermediate files required for processing data with ArcGIS are obtained. Notably, ArcGIS was also developed for the characterization of drainage systems (stream networks, watersheds, floodplain characteristics) and model integration. The structure of ArcGIS has extensibility through tools composed of modules or scripts written in Python (High-level programming language) and is integrable to the central module of ArcGIS. ArcGIS is a proprietary commercial and licensed software package for the development and manipulation of vector and matrix information for the use and management of thematic bases. ArcGIS provides a GIS (geographic i*nformation system) environment* with a range of tools in an integrated way and good software usability. Geoprocessing can be understood as the technical and conceptual linking of tools for capturing, storing, and processing data, as well as presenting georeferenced spatial information.

The WRF-Hydro GIS preprocessing toolkit was designed by NCAR [44] to facilitate the process of the derivation of input files and WRF-Hydro parameters from commonly available geospatial data products, such as digital elevation models, land use and occupancy, geospatial metadata to support georeferencing of relevant WRF-Hydro model output files, and shapefiles to help visualize model components.

WRF-Hydro GIS preprocessing tools are designed to function as an additional Python toolbox in the ArcGIS ESRI software. The specific operating system and software requirements are covered by the complete documentation of the WRF-Hydro GIS preprocessing toolkit [44].

As mentioned, working with the WRF-Hydro system requires a series of steps that may present some complexity for researchers from different areas but that are fundamental in the study of the hydrological aspects of a hydrological basin. Information on the study of water is needed for decision-making in several studies, which are often scarce, preventing adequate management of water resources, as mentioned in the Hydrological Atlas of the Rio Grande River basin [40].

Thus, construction of a web tool that minimizes preparation of specific computational codes capable of intuitively integrating the execution steps of WRF-Hydro is an alternative for engineers, scientists, and other professionals related to the area of hydrometeorology. This alternative allows them to develop their studies with quality equivalent to the conventional work of WRF-Hydro, without the need for possible assistance from a computer professional, thus stimulating the increase in research in this area and overcoming potential failures in the hydrological monitoring of basins.

### Hydrometeorological modeling with AI approach

Agile neural networks that can be used in this solution include *convolutional neural* networks (CNNs) and recurrent networks called long short-term memory (LSTM). A brief overview of these types of neural networks will be shown in this section.

#### Convolutional Neural Networks (CNNs)

A CNN is a type of artificial neural network that applies filters to input data and has great learning power for spatial and even time relationships within the input data. Neural networks are computational models inspired by the central nervous system of an animal that can perform machine learning as well as pattern recognition. Additionally, CNNs have a *pooling layer to* reduce the necessary processing of network training. This layer is responsible for reducing the spatial size of the convolution step output. Examples of operations of this type are the maximum value (MaxPooling) and the average value (AvgPooling) in each fixed-size window. Then, there is a layer responsible for leveling the output to a one-dimensional layer, the output of which is eventually submitted to the last part of a CNN: one or more fully connected layers, or multilayer perceptron (MLP) [45], which performs the final classification or regression task. Because of these characteristics, CNNs are widely used in the area of computer vision to perform tasks, such as detecting objects in images [24, 46]. Fully convolutional networks *(*FCNs) are a type of convolutional neural network that do not have the final layer of MLP and extract the main characteristics of degrees from the data for use in subsequent layers, such as those in hybrid approaches.

#### Long-term Neural Memory Networks (LSTM)

LSTM networks are a special type of neural recurrent network that have been created as an approach to the problem of forgetting long-term dependencies common in recurrent neural networks. The LSTM network was first introduced by [47], and since then, many variants of this neural network have been created.

**Fig. 4:**
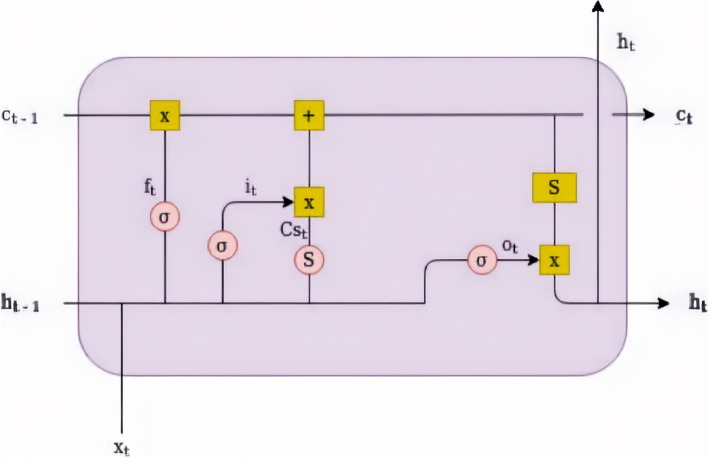
Structure of an LSTM cell. Source: A figure adapted from [46]

Figure 4 illustrates the structure of an LSTM cell, in which arrows represent the data flow, and squares, point operations, and circles correspond to activation functions. The main part of an LSTM network is the state of the cell, which is an internal selective memory of the past, represented by the horizontal line that starts at c_t-1_ and ends at c_t._ The hidden state, represented by h, is the output of the LSTM cell.

## Results and discussion

### WRF-Hydro and ArcGIS integrated with API in a web interface

Based on the analyses carried out in a multidisciplinary research environment, it was possible to obtain information about the quality, accuracy, and integration of WRF-Hydro tools with the respective integration modules in an HPC (High-Performance Computing) environment, ESRI ArcGIS as an independent layer, and a web interface.

From the nonintuitive processes in which this information was obtained, the proposal of the web interface is considered. Some authors highlight the important characteristics that software usability offers us, associating the ease with which new users can initiate effective interaction and achieve maximum performance in tasks that are included in the context of [48, 49].

From this perspective, an integration structure of WRF-Hydro and ArcGIS is presented through a web usability tool suitable for researchers or potential customers through the visualization and operation of simulation processes to monitor and perform predictability analyses of hydrometeorological variables in each region.

**Fig. 5:**
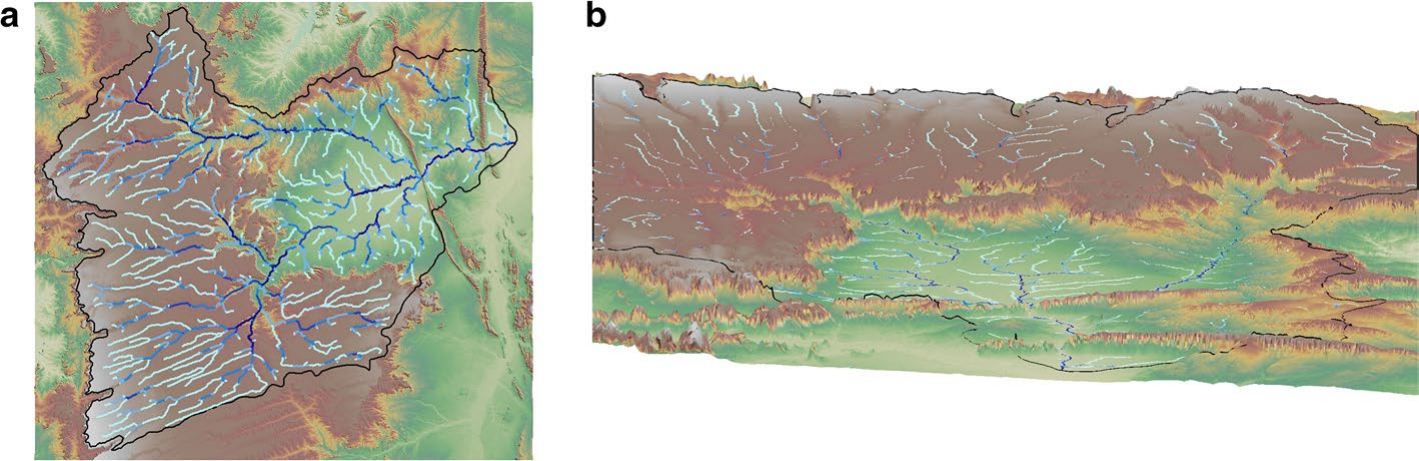
Flow of the Rio Grande DEM stream (**a**) and Rio Grande DEM 3D (**b**)

Figure 5 represents a digital elevation model (DEM) with river flows in the upper view. Video 01 represents a DEM with the simulation of river flows in an animation that exposes more details about this micro basin. To access this animation, click the animation hyperlink or visit this URL (Video 01, https://youtu.be/oQiw9nHedtg).

**Fig. 6:**
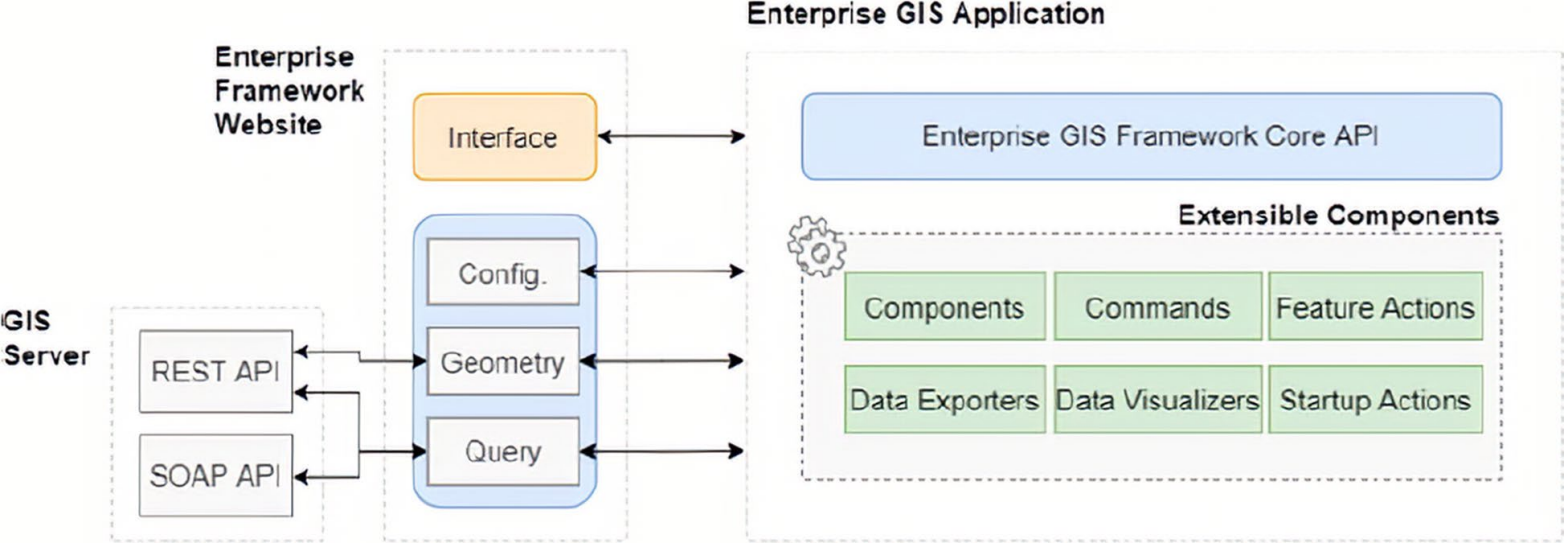
ArcGIS Framework. Source: A figure adapted from [50]

These results were generated with a multidisciplinary team with distinct functions, which can be simplified into a single GIS web tool that provides a single view of the dashboard designed based on software usability with a native integration, as shown in Fig. 6, through the *Rest* architecture, SOAP protocol (S*imple Object Access Protocol),* and API (Application Programming Interface), which was developed in programming languages, such as Java.NET and Python.

**Fig. 7:**
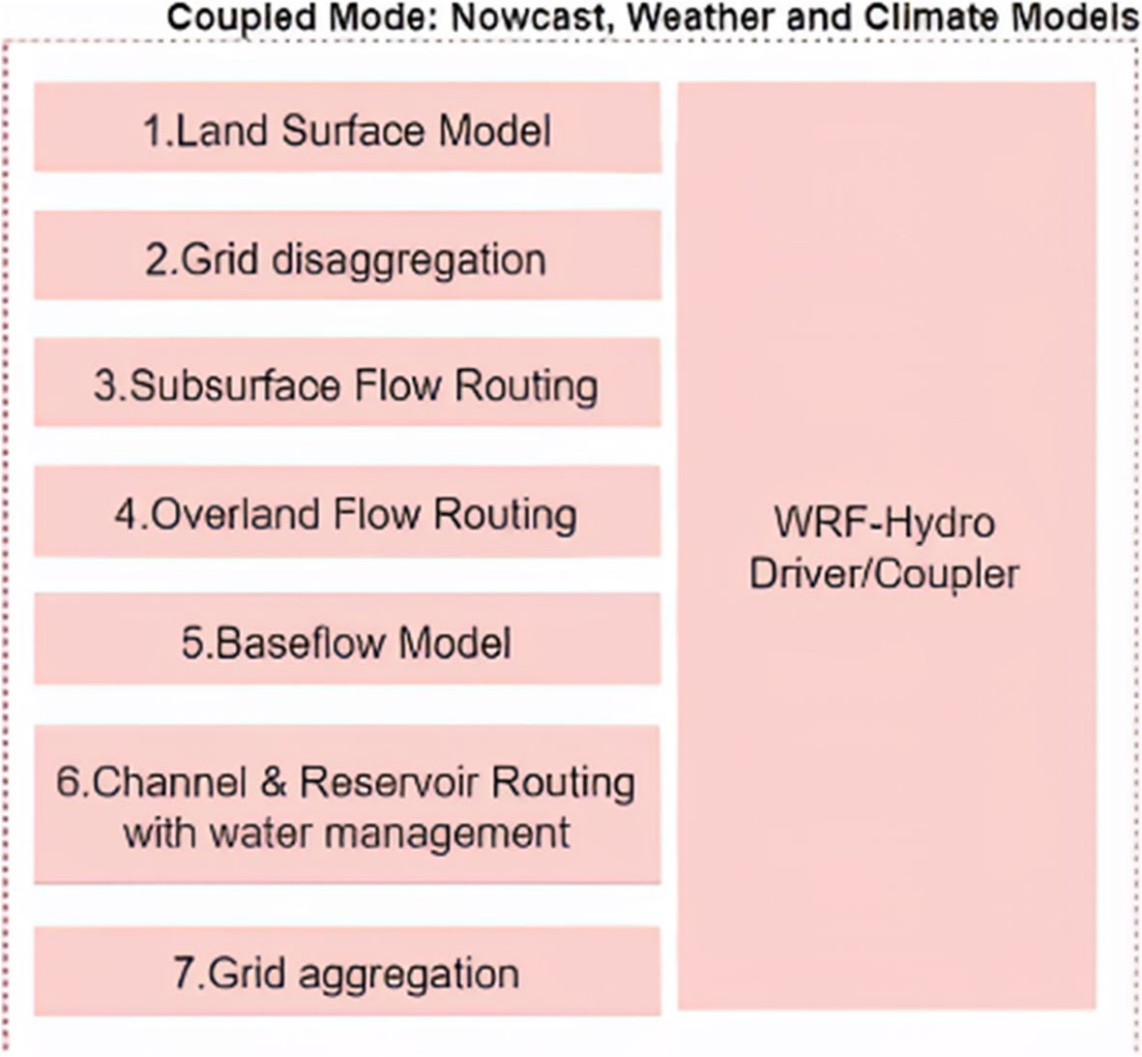
WRF-Hydro Structure. Source: A figure adapted from [7]

Figure 7 displays the WRF-Hydro Coupled Model*,* which is commonly loaded in the HPC environment and can be operated by automated Python scripts for simulation routines.

**Fig. 8:**
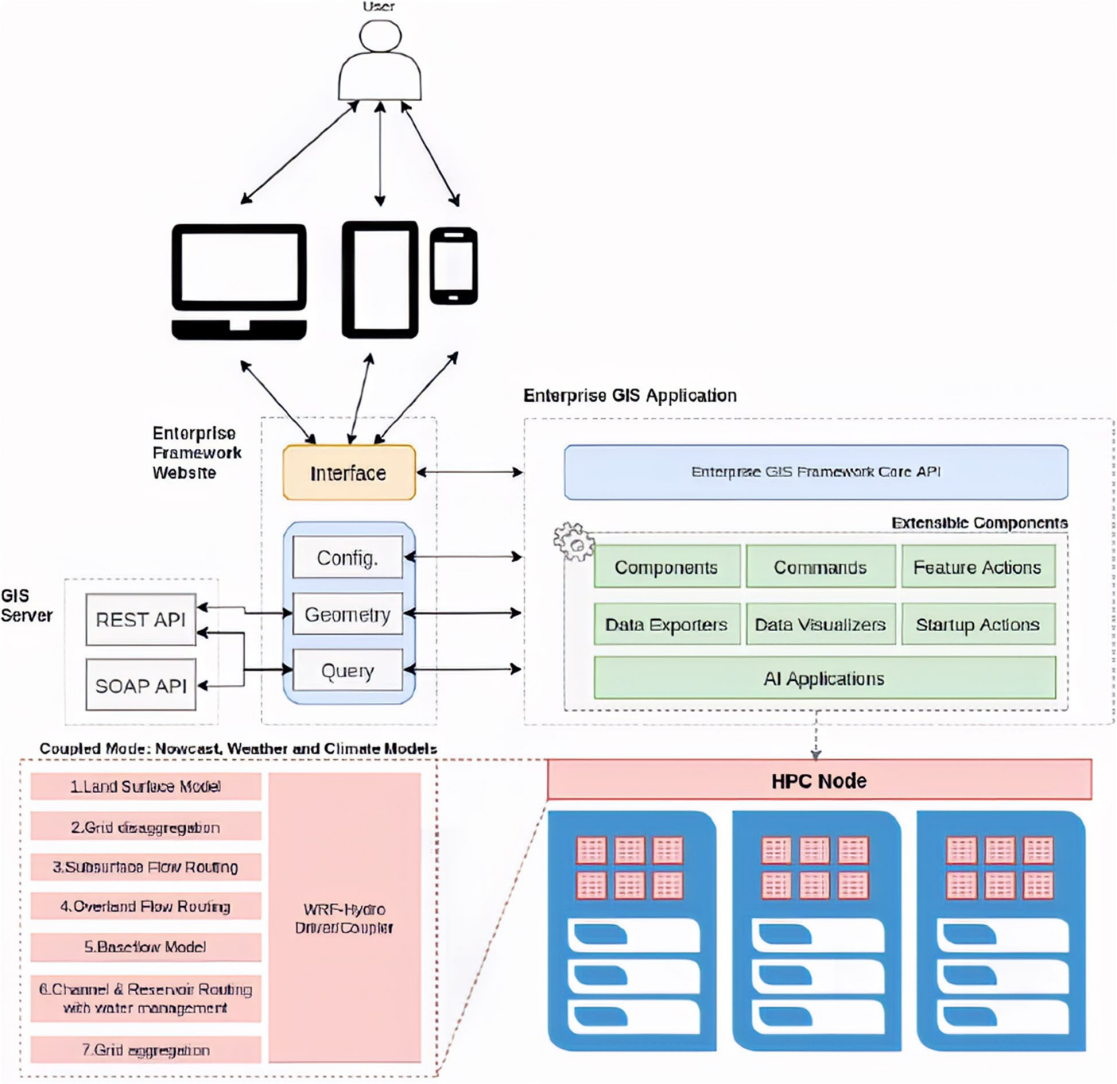
WRF-Hydro, ArcGIS, and AI integration structure. Source: A figure adapted from [7, 50]

Figure 8 shows a representation of the logical integration of solutions using a GIS web tool.

### AI application module

Although WRF-Hydro-related tools are considered to be state-of-the-art in the context of hydrometeorological modeling, some of the significant limitations are computational cost and simulation time required. Additionally, model calibration (optimization of model parameters for better simulation of the different physical processes) is an arduous task that requires good surface and startup data, which is often scarce or not suitable for forecasting applications. Once the calibration has adjusted and the results stabilize, the model can be directed in a particular way to rescan for reliable results within their respective limits. Thus, it is possible to provide greater accuracy; but there is still a considerable effort require in the configuration, and a long period may be required to obtain good results, which is also restricted to a defined period.

At this point, the application of AI algorithms can overcome some of these barriers. Good results have been found with applications of neural networks for simulations, in short- and medium-term periods [46], and for concentrations of pollutants [46], and applications can be extended to the field of hydrometeorology. These models will also be costly during the training process; however, assuming a sufficiently large amount of data and with proper processing of features*,* these models can be generalized, which demonstrates good accuracy and no restrictions on a given period. Thus, it is possible to integrate an AI module into the architecture proposed in [51] that contains previously trained models; it is also possible to manage algorithms to validate and compare studies between these approaches and the WRF-Hydro model results over short and medium-term periods, providing knowledge for long-term evaluations and optimizing the cost and time use. In addition, obtaining the results more systematically will serve as a basis for better decision making in river basin management and more efficiently and quickly forecasting extreme events (droughts and floods). Additionally, the management of water bodies can help with contamination indicators for the SARS-CoV-2 pandemic and is an additional tool for the monitoring of wastewater and water bodies. In particular, this technique concerns the association of pandemic indicators with the characteristics of the water body, which is crucial information modeled by WRF-Hydro and optimized by AI.

## Conclusion

The research is being developed by an interdisciplinary group of researchers who are working on executing all the steps necessary to conduct hydrometeorological simulations for the MATOPIBA region. Regional geoprocessing was completed using ArcGIS, and partial results of the detailed regional information were obtained, which is important for the next stages of the study. In addition, flow, precipitation, and other variables were obtained, demonstrating that the WRF-Hydro system is an efficient hydrometeorological simulation model. Thus, a system with AI tools is promising to reduce the computational cost and may be suitable for the analysis of water bodies and possible indices of contagion evolution by SARS-CoV-2 with monitoring and mitigation of the number of cases related to the current pandemic. A significant advancement has been made in the processing and application of the WRF-Hydro model, and the research group is still working on some steps to improve simulation accuracy through automated and efficient data calibration.

Therefore, with the consolidation of the described processes, we seek to implement the proposed solution, which is feasible, especially given the significant number of steps required for monitoring hydrometeorological variables, and this process was largely carried out by scholars who were not experts in the area of computing. Thus, this study provides excellent prospects for the use of AI as a tool for the traditional management of water resources and as a solution capable of correlating water management with indicators of contamination in pandemics, such as SARS-CoV-2, which we currently face.

## Data Availability

All data generated or analyzed during this study are included in this published article.

## Abbreviations

AI: Artificial Intelligence
API: Application Programming Interface
ArcGIS: Geographic Information System
AvgPooling: Average pooling value
CNN: Convolutional Neural Networks
COVID-19: Coronavirus Disease 2019
cpl: Coupling Interface
c_t_: C represents the cell state at a given timestep t
DEM: Digital Elevation Model
Drv: Driver
D03: Domain 03
ESRI: Environmental Systems Research Institute
FCN: Fully Convolutional Networks
GDAS-FNL: Global Data Assimilation System Final Analysis
h: Hour
HPC: High Performance Computing
h_t_: H represents the output of the LSTM block at a given timestep t
MATOPIBA: Acronym for Maranhão - Tocantins - Piauí - Bahia
MaxPooling: Maximum polling value
MLP: Multilayer Perceptron
NASA: National Aeronautics and Space Administration
NCAR: National Center for Atmospheric Research
NCEP: National Centers for Environmental Prediction
LSTM: Long Short-Term Memory Neural Networks
REST: Representational State Transfer
SARS-CoV-2: Severe Acute Respiratory Syndrome Coronavirus 2
s: seconds
SIG: Geographic Information System
SOAP: Simple Object Access Protocol
USGS: United States Geological Survey
UTC: Coordinated Universal Time
WBE: Wastewater-Based Epidemiology
WRF: Weather Research and Forecasting
WRF-Hydro: Weather Research and Forecasting – Hydro
